# OmniCorr: an R-package for visualizing putative host-microbiome interactions using multi-omics data

**DOI:** 10.1093/bioadv/vbag057

**Published:** 2026-02-17

**Authors:** Shashank Gupta, Veronica Quarato, Wanxin Lai, Carl M Kobel, Velma T E Aho, Arturo Vera-Ponce de León, Sabina Leanti La Rosa, Simen R Sandve, Phillip B Pope, Torgeir R Hvidsten

**Affiliations:** Faculty of Chemistry, Biotechnology and Food Science, Norwegian University of Life Sciences, Ås, Norway; Faculty of Chemistry, Biotechnology and Food Science, Norwegian University of Life Sciences, Ås, Norway; Faculty of Chemistry, Biotechnology and Food Science, Norwegian University of Life Sciences, Ås, Norway; Faculty of Biosciences, Norwegian University of Life Sciences, Ås, Norway; Faculty of Biosciences, Norwegian University of Life Sciences, Ås, Norway; Faculty of Biosciences, Norwegian University of Life Sciences, Ås, Norway; Faculty of Chemistry, Biotechnology and Food Science, Norwegian University of Life Sciences, Ås, Norway; Faculty of Biosciences, Norwegian University of Life Sciences, Ås, Norway; Faculty of Chemistry, Biotechnology and Food Science, Norwegian University of Life Sciences, Ås, Norway; Faculty of Biosciences, Norwegian University of Life Sciences, Ås, Norway; Centre for Microbiome Research, School of Biomedical Sciences, Queensland University of Technology (QUT), Translational Research Institute, Woolloongabba, Queensland, Australia; Faculty of Chemistry, Biotechnology and Food Science, Norwegian University of Life Sciences, Ås, Norway

## Abstract

Holo-omics leverages omics datasets to explore the interactions between hosts and their associated microbiomes. Although the generation of omics data from matching host and microbiome samples is steadily increasing, there remains a scarcity of computational tools capable of integrating and visualizing this data to facilitate the prediction and interpretation of host-microbiome interactions. We present **OmniCorr**, an R package designed to: (i) manage the complexity of omics data by clustering co-varying features (e.g. genes, proteins, and metabolites) into modules, (ii) visualize correlations of these modules across different omics layers, host-microbiome interfaces, and metadata, and (iii) identify statistically significant associations indicative of putative host-microbiome interactions. OmniCorr’s utility is demonstrated using datasets from two systems: (i) Atlantic salmon, integrating host transcriptomics with metagenomics and metatranscriptomics to explore dietary impacts, and (ii) cattle, combining host proteomics with metaproteomics to investigate methane emission variability.

*Availability and implementation*: OmniCorr is freely available at https://github.com/shashank-KU/OmniCorr.

## 1 Introduction

The holobiont, comprising a host organism and its associated microbiome, represents a complex biological system with intricate interactions that significantly influence health and disease states (Bordenstein and Theis 2015, Robinson and Cameron 2020). These host-microbiome interactions play crucial roles in various physiological processes, including metabolism, immune function, and overall well-being (Bordenstein and Theis 2015, Rowland *et al*. 2018). Multi-omics is increasingly being used for molecular profiling of matching samples from host tissues and gut microbiota collected, e.g. during feeding trials designed to modulate the gut microbiota or across individual animals with variations in physiological traits believed to be affected by the gut microbiota (Kwoji *et al*. 2023, Muller *et al*. 2024).

The aim of holo-omics studies is to identify putative interactions between molecular markers in the host and the microbiome that could confer an effect on the host (Nyholm *et al*. 2020). However, discerning true host-microbiome associations from those driven by random variability or single outlier features remains challenging. The multi-omics datasets generated from holo-omics studies present several data analysis challenges, one of which is the large number of features (genes, proteins, metabolites, microbial species: ranging from thousands to millions) compared to the relatively small number of samples (ranging from tens to a few hundreds) (Zhao *et al*. 2019). Several computational methods have been proposed (Kobel *et al*. 2024), mostly adapting existing multi-omics tools. Latent factor modelling approaches such as MOFA, mixOmics and Holomics (Rohart *et al*. 2017, Argelaguet *et al*. 2020, Munk *et al*. 2024) dominate current practice. For a comprehensive review of latent variable and related multi-omics integration methods, see Baião *et al*. (2025). However, when features vastly outnumber samples (*P* ≫ *n*), factor models are weakly constrained and must estimate thousands of loadings from only dozens of samples, which increases the risk of overfitting. These methods also yield latent factors that do not map directly to the original features, requiring post-hoc decoding to link them back to specific genes, proteins, or metabolites.

OmniCorr takes a different approach: it first groups features into co-varying modules and then directly correlates module representatives across omics layers and across the host–microbiome boundary. This reduction in dimensionality makes the data amenable to more robust statistics and reduces the burden of multiple testing, enabling discovery. Using representatives of modules also yields named, biologically coherent units that map directly to genes/proteins/taxa or to enriched functions/pathways, enabling biological interpretation. Here we present OmniCorr as an easy-to-use R package for exploratory holo-omics analysis. It provides a streamlined workflow with flexible visualization that complements more complex integrative frameworks, particularly in studies with limited sample sizes and a need for interpretable host–microbiome associations.

## 2 Methods

The OmniCorr method approaches the challenge of predicting host-microbiome interactions from omics data by first reducing the dimensionality of the omics data and then by visualizing correlations across omics-layers, host-microbiome boundaries and between omics data and metadata.

The package can be installed with the command:

devtools*::*install_github(“shashank-KU/OmniCorr”)

and consists of the following steps:

**Step 0: Reduce dimensionality of omics data**: This step allows the user to choose their preferred methods for preprocessing and module detection.

As OmniCorr is designed to be agnostic to specific omics types, it does not impose specific preprocessing steps designed for e.g. sparse or compositional data. Instead, users should preprocess these layers according to best practices, e.g. filtering low-prevalence features and normalizing using methods such as centered log-ratio (CLR) transformation, cumulative sum scaling (CSS) or variance-stabilizing transformation (VST). This modularity allows users to tailor preprocessing to the statistical properties of their data before applying OmniCorr for integrative correlation analysis.

For module detection, we recommend the robust inference of network modules as implemented in the R package Weighted Gene Co-expression Network Analysis (WGCNA) (Langfelder and Horvath 2008). Here, the function blockwiseModules identifies modules and the function chooseTopHubInEachModule extracts the most connected gene, protein or metabolite (hub) from each of these modules. This hub selection is based on intramodular connectivity, as implemented in the WGCNA package, where it has been shown to produce biologically meaningful and robust module representatives (Langfelder and Horvath 2008). Although other representations such as eigengenes or centroids may be used, the default selection of the top hub is grounded in the reproducibility and interpretability of WGCNA-defined hubs across diverse datasets. The output from step 0 is thus a table for each omics layer with samples as rows (these samples must match across all omics layers and species/kingdom) and hubs as columns.

**Step 1: Perform hierarchical clustering of the central omics layer**: For the visualization, the user selects one central omics layer where the profiles of the hubs across samples will be displayed as a heatmap. The other omics layers will appear in the visualization only as hub-hub-correlations. The hubs in the chosen omics layer are ordered using hierarchical clustering (hclust-function).

**Step 2: Generate a heatmap of the central omics layer with dendrogram**: Given the hierarchical clustering from Step 1 (i.e. the dendrogram), a heatmap is generated for the central omics layer using the pheatmap-function.

**Step 3: Calculate correlations between omics data**: The OmniCorr function calculate_correlations computes correlations between the modules (represented by one hub per module) in the central omics layer and the modules of other omics layers. If the centrepiece of the visualization is from the host, the other layers are typically from the microbiota. The correlations indicate to what degree modules in the central layer co-vary across the samples with the modules of each of the other layers. Significance for the correlations can also be computed and added to downstream correlation heatmaps. The output is a module-module correlation matrix per omics layer.

To provide flexibility and transparency in statistical analysis, the calculate_correlations() function allows users to specify the correlation method (“pearson”, “spearman”, or “kendall”), handle missing data using various approaches (e.g. “all.obs”, “pairwise.complete.obs”), and choose the method for adjusting *P*-values for multiple testing (e.g. “fdr”, “bonferroni”). The function computes correlation coefficients between all feature pairs from the two input omics layers, tests for statistical significance, and returns matrices of correlation values, raw *P*-values, adjusted *P*-values, and annotated significance levels. Significance can be reported as asterisks (e.g. *, **, and ***), exact *P*-values, or raw correlation values, depending on user preference. This enables users to tailor the analysis to the context of their specific dataset and to interpret correlations with statistical confidence.

**Step 4: Generate a heatmap of the correlations between the central layer and other layers**: Heatmaps for correlations from Step 3 are generated using the pheatmap-function.

**Step 5: Integrate the metadata heatmap:** Finally, the OmniCorr function calculate_correlations is used to compute correlations between the modules in the central omics layer and metadata. The correlations are represented as a heatmap, annotated with significant associations, and the metadata correlation heatmap is combined with the heatmaps from Step 5 to display an integrated overview of correlations across omics layers and host-microbiome boundaries.

**Additional analysis**: Other tools such as the R package clusterProfiler (Yu *et al*. 2012) can be used to perform Gene Ontology (GO) and pathway enrichment analysis of the modules. Enriched functions, pathways or bacterial phyla can be used to name modules in the visualization in order to enhance interpretation. Enrichment analysis methods can vary depending on the omics layer (e.g. microbial species, genes, and proteins), and the outputs of OmniCorr are structured for compatibility with standard enrichment packages.

## 3 Results

### 3.1 Case study 1: Feed-host-microbiome interaction in Atlantic salmon

**Figure 1:**
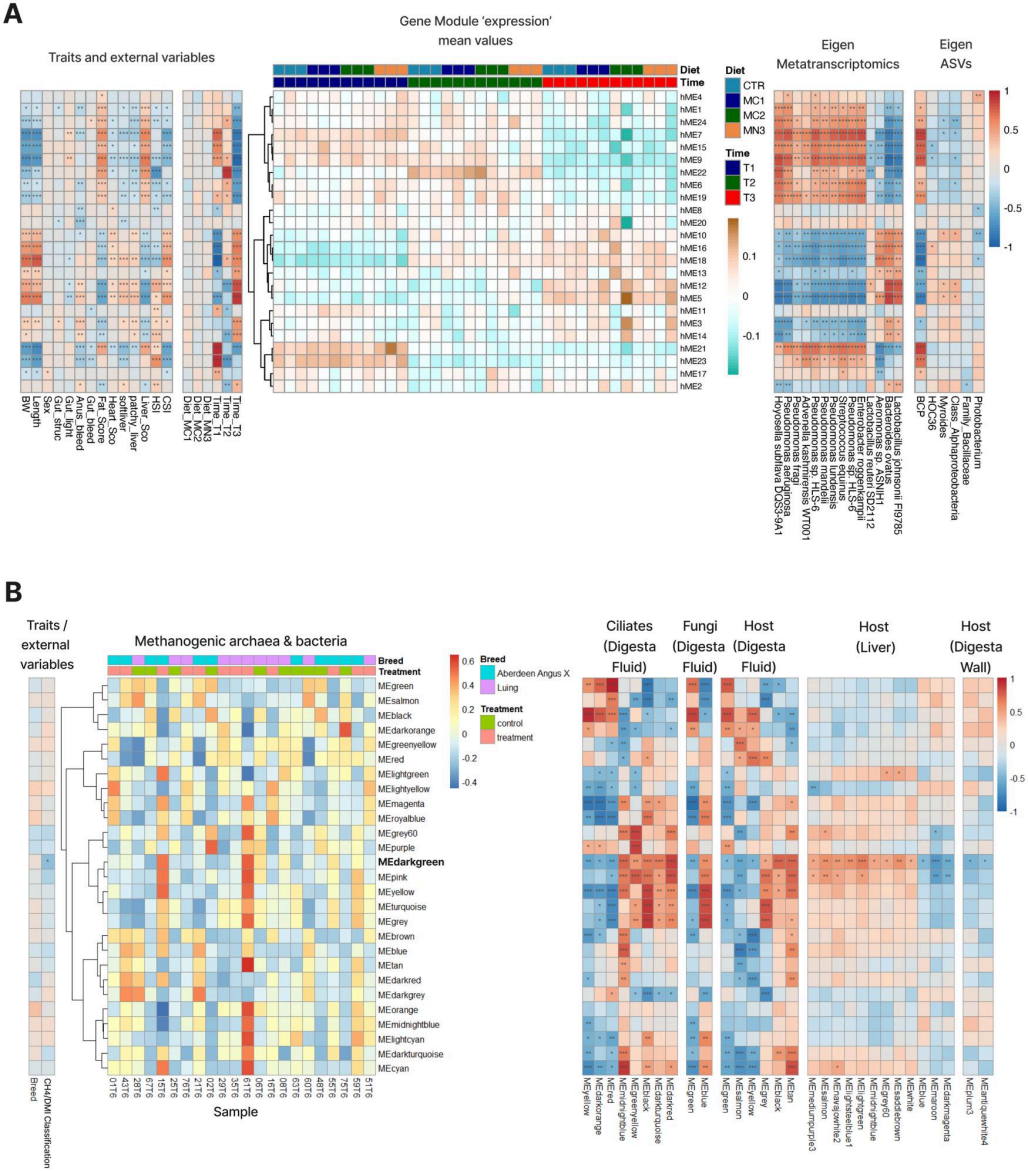
Two case studies applying OmniCorr to multi-omics data from (A) Atlantic salmon and (B) cattle. The central heatmap visualizes the central omics layer with columns corresponding to samples and rows corresponding to modules. Correlations between the central omics layer and metadata are visualized to the left, with stars indicating significant correlations. Correlations between the central omics layer and modules in other omics layers are visualized to the right.

Microbiome-directed dietary interventions, such as microbiota-directed fibres (MDFs), have been effective in promoting beneficial gut microbes in several animal production systems. However, there are knowledge gaps related to the specific metabolic influence that such feed supplements exert in their host and/or their microbiota. To investigate the effects of dietary supplementation on Atlantic salmon (*Salmo salar*), Gupta et al. (Gupta *et al*. 2024) conducted a feeding trial designed to assess individualized responses to an industry-standard 0.2% inclusion of one acetylated β-galactoglucomannan (MN3) and two different types of α-mannans (MC1 and MC2), in comparison to a standard diet (CTR). This trial lasted for 110 days, transitioning from freshwater to seawater, with samples collected at four distinct time points (T0, T1, T2, and T3). The omics analysis included host transcriptomics from the hindgut (69 389 genes), microbial community characterization through 16S rRNA gene amplicon sequencing (6468 Amplicon Sequencing Variants—ASVs-identified), and metatranscriptomics (117 261 genes). Additionally, the collected metadata comprised measurements of fish weight, length, gutted weight, condition factor, hepatosomatic index, cardio somatic index, welfare indicators and organ integrity (Gupta *et al*. 2024). In the present study, we applied OmniCorr to this dataset, while excluding the samples from the T0 sampling time point (as no experimental diet was provided during that period). We reduced the dimensionality of the omics data and identified 24 host modules, 6 microbial modules, and 14 metatranscriptome modules. Several host and microbiota modules were correlated with sampling time points, whereas no effect was detected for the feed additive mannan (Fig. 1A), in agreement with our previous results (Gupta *et al*. 2024).

Host modules hME9 and hME5 demonstrated strong correlations with the water environment (fresh vs. salt). Correlations between the host omics data and water environment changes is expected in this dataset as Atlantic salmon undergoes complex preparatory physiological changes to an adult life in seawater and experience additional acute physiological responses when salinity levels increase (Harvey *et al*. 2024). Furthermore, the host module (hME9) was strongly and significantly positively correlated with ASVs from the *Burkholderia-Caballeronia-Paraburkholderia* (BCP) group, suggesting a close relationship between the host’s molecular responses and specific microbial communities. This correlation highlights potential host-microbe interactions, where the identified microbial taxa may play supportive roles in facilitating host adaptation during the transition to seawater. In the BCP group, we identified genera such as *Pseudomonas*, which were also detected in the metatranscriptomics data and exhibited strong positive correlations with host module (hME9) (Fig. 1A). Moreover, manual inspection of gene expression by BCP taxa in Gupta et al. (Gupta *et al*. 2024) highlighted the metabolic influence this group can exert towards digestion and generation of energy-yielding nutrients.

### 3.2 Case study 2: Methane emission in different cattle breeds

Livestock significantly contribute to global greenhouse gas emissions, particularly methane (Blaustein-Rejto *et al*. 2023), which is produced during digestion in the rumen where complex microbial communities reside (Grossi *et al*. 2019). Methane is a by-product of methanogenesis, a process driven by methanogenic archaea. While bacteria do not directly participate in methanogenesis, they break down complex carbohydrates, producing by-products such as hydrogen, which fuel other microbes and support methanogens. Understanding the interactions between the host and its microbiome can therefore help reduce emissions by pinpointing microbial activities that can be targeted without affecting livestock productivity. In this study, we integrated (meta)proteomics data from multiple layers of the rumen ecosystem: methanogenic archaea, bacteria, ciliate protozoa, and fungi (all from digesta fluid samples), as well as host tissues (digesta fluid, liver, and rumen wall) (Kobel *et al*. 2025). Samples were collected from twenty-two cattle (Luing and Aberdeen Angus ×), kept under identical conditions and classified as high- or low-methane emitters based on respiration-chamber measurements (above or below 24 CH_4_ [g/kg DMI]).

We used methanogenic Archaea and Bacteria (ArcBac) as the central omic layer to explore interspecies interactions that influence methane emissions. We applied OmniCorr and identified 27 modules from ArcBac, 31 from ciliates (6473 proteins), and 7 from fungi (82 proteins), respectively. In the host, we found 15 modules (222 proteins) from digesta fluid, 68 modules (2389 proteins) from liver, and 92 modules (2619 proteins) from the digesta wall (Fig. 1B). Notably, the darkgreen module from ArcBac showed a significant correlation with methane emission and was also correlated with modules from ciliates (8), fungi (2), host digesta fluid (6), liver (12), and rumen wall (2). The darkgreen module, enriched for enzymes involved in streptomycin biosynthesis, contained 30 proteins with lower abundance in high methane emitters compared to low emitters (Figs 1 and 2, available as supplementary data at *Bioinformatics Advances* online). How elevated streptomycin production might influence the ruminal metabolic networks that drive methanogenesis is a tantalizing question, but is supported by evidence that antibiotic use (e.g. monensin) can effectively reduce methane emissions in cattle (Appuhamy *et al*. 2013). Moreover, strong correlations between this module and ciliate modules enriched in KEGG pathways related to organismal systems and metabolism (Fig. 1, available as supplementary data at *Bioinformatics Advances* online) suggests that ciliates modulate their archaeal and bacterial partners, coordinating complex symbiotic interactions within metabolic networks (Rossi *et al*. 2019, Kobel *et al*. 2025). Cross-domain interaction between the ArcBac and fungi was observed for the blue and green modules, which were enriched in nitrogen metabolism. This finding suggests the influence of fungi in shaping the microbial community by altering the availability of ammonia or nitrates and hydrogen, which has an indirect effect on methanogens (Yang *et al*. 2016). Importantly, some host modules also demonstrated significant correlations with both methane emission levels and the darkgreen module from ArcBac. The black module, enriched for G protein-coupled receptors (GPCRs), opens future hypotheses to test if these proteins in the cattle gut could contribute to physiological functions that impact digestion and metabolic efficiency. Additionally, the enrichment of pathways in the black module related to serotonergic synapse, neuroactive ligand-receptor interactions, and axon regeneration are in line with previous findings reporting that neurotransmitters in the enteric nervous system modulate gut mobility, secretion, and the microbial community in response to stress signaling (Layunta *et al*. 2021,  Stepniewski *et al*. 2021). Finally, the midnightblue module from the cattle liver was enriched for pathways highly relevant to immune and inflammatory responses: phagosome, peroxisome, endocytosis, and neutrophil extracellular trap formation. Both these modules (black and midnightblue) contained proteins with higher abundance in low emission cattle compared to high emission cattle (Li *et al*. 2023, Ma *et al*. 2024).

### 3.3 Comparison to existing multi-omics integrators

Several computational methods have been developed for integrating multi-omics data (for a comprehensive overview see https://github.com/mikelove/awesome-multi-omics), but few have been tailored to holo-omics data spanning the host-microbiome boundary. Here, we focus on factor-based approaches because they currently dominate the landscape of integrative omics tools.

Holo-omics data sets typically contain far more features than samples (thousands of genes, proteins, metabolites vs. dozens of samples). In such *P* ≫ *n* settings, factor-based methods are easily under-constrained and prone to overfitting—a classic “curse of dimensionality.” Factor models such as MOFA mitigate this by learning a small set of latent factors with Bayesian sparsity priors and variational inference. However, they still estimate thousands of loadings from only tens of samples, which can yield unstable or hard-to-interpret factors.

OmniCorr takes the reverse route: it first aggregates features into biologically coherent modules and then correlates module representatives across layers and metadata. This shrinks the parameter space, reduces the burden of multiple testing, and keeps the biological units explicit—improving stability and interpretability in the small-sample settings typical of host–microbiome studies. To illustrate this, we re-analysed the cattle (meta)proteomics dataset using MOFA under like-for-like preprocessing (Fig. 3, available as supplementary data at *Bioinformatics Advances* online). While OmniCorr recovered a methane-associated module of methanogenic Archaea and bacteria, none of the MOFA factors showed a significant correlation with methane. One factor carried high loadings for streptomycin-biosynthesis enzymes, but this factor explained variance specific to the liver proteome only. Taken together, this illustrates how a module-first strategy can recover biologically meaningful structure that factor models fail to capture in small-sample holo-omics settings.

## 4 Discussion

We showcase two examples of vastly different animal host-gut microbiome systems, where OmniCorr can unveil possible host-microbiota interactions that could be tested in future analyses. Although the Atlantic salmon feeding trial revealed no effect of adding 0.2% mannan to the diet (Gupta *et al*. 2024), the analysis highlighted several other potentially interesting correlations across the host-microbiota axis.

The cattle-emission analysis based on the data from Kobel et al. (Kobel *et al*. 2025) revealed a network of 22 key modules across microbial- and host-derived proteins linked to methane emission. Our results are in line with previous findings that have shown that “high methane emission” rumina prioritize energy harvesting and are dominated by methanogens and their associated fermentative bacteria (Shabat *et al*. 2016, Wang *et al*. 2017, Pereira *et al*. 2022, Khairunisa *et al*. 2023). Meanwhile, the enriched pathways identified from host-associated modules were related to neurotransmission, inflammatory responses, and cellular signalling, proposing a role of the host in coordinating microbial activity and digestion, which contributes to altered metabolic efficiency. Understanding these interactions may offer insights into how systemic physiological responses, influenced by both host and microbial factors, contribute to methane emission.

To address the robustness of associations identified by OmniCorr, we evaluated their biological plausibility in both case studies. For example, in the Atlantic salmon dataset, the correlation between host module hME9 and the *Burkholderia-Caballeronia-Paraburkholderia* group is consistent with known roles of these taxa in nutrient metabolism and adaptation to saltwater environments. Similarly, in cattle, the ArcBac darkgreen module correlated with methane emission and was enriched for streptomycin biosynthesis—a pathway previously implicated in methane suppression via antibiotic use (Appuhamy *et al*. 2013). These findings suggest that OmniCorr captures biologically meaningful signals.

In our methane case study, OmniCorr recovered a methane-associated module that was not detected by factor analysis, illustrating the practical benefit of this approach. We recognize that tools such as mixOmics and MOFA have advanced the field of multi-omics integration by providing latent variable models capable of explaining shared variance across datasets. However, these methods may be less accessible to users focused on transparent interpretation and hypothesis generation from limited sample sizes. OmniCorr is designed to complement such tools by providing a user-friendly, statistically transparent workflow for visualizing significant cross-omics correlations. It serves as a platform to pinpoint interpretable associations that warrant further validation and explicitly offers solutions to analyze host-microbiota systems.

In conclusion, while omics layers can be analyzed separately with respect to e.g. dietary effects, we show that using the OmniCorr package, new insight can be gained from an integrated analysis of the host and its associated microbiome. Our computational approach enables analysis at the holobiont level, explicitly capturing the interplay between the host and its associated microbiome.

## Supplementary Material

vbag057_Supplementary_Data

## Data Availability

The original data and code are available at https://github.com/shashank-KU/OmniCorr.

## References

[vbag057-B1] Appuhamy JADRN , Strathe AB, Jayasundara S et al Anti-methanogenic effects of monensin in dairy and beef cattle: a meta-analysis. J Dairy Sci 2013;96:5161–73. 23769353 10.3168/jds.2012-5923

[vbag057-B2] Argelaguet R , Arnol D, Bredikhin D et al MOFA+: a statistical framework for comprehensive integration of multi-modal single-cell data. Genome Biol 2020;21:111. 32393329 10.1186/s13059-020-02015-1 PMC7212577

[vbag057-B3] Baião AR , Cai Z, Poulos RC et al A technical review of multi-omics data integration methods: from classical statistical to deep generative approaches. Brief Bioinform 2025;26:bbaf355. 40748323 10.1093/bib/bbaf355 PMC12315550

[vbag057-B4] Blaustein-Rejto D , Soltis N, Blomqvist L. Carbon opportunity cost increases carbon footprint advantage of grain-finished beef. PLoS One 2023;18:e0295035. 38091302 10.1371/journal.pone.0295035 PMC10718409

[vbag057-B5] Bordenstein SR , Theis KR. Host biology in light of the microbiome: ten principles of holobionts and hologenomes. PLoS Biol 2015;13:e1002226. 26284777 10.1371/journal.pbio.1002226 PMC4540581

[vbag057-B6] Grossi G , Goglio P, Vitali A et al Livestock and climate change: impact of livestock on climate and mitigation strategies. Anim Front 2019;9:69–76. 10.1093/af/vfy034 PMC7015462 32071797

[vbag057-B7] Gupta S , Vera-Ponce de León A, Kodama M et al The need for high-resolution gut microbiome characterization to design efficient strategies for sustainable aquaculture production. Commun Biol 2024;7:1391. 39455736 10.1038/s42003-024-07087-4 PMC11511968

[vbag057-B8] Harvey TN , Gillard GB, Røsæg LL et al The genome regulatory landscape of atlantic salmon liver through smoltification. PLoS One 2024;19:e0302388. 38648207 10.1371/journal.pone.0302388 PMC11034671

[vbag057-B9] Khairunisa BH , Heryakusuma C, Ike K et al Evolving understanding of rumen methanogen ecophysiology. Front Microbiol 2023;14:1296008. 38029083 10.3389/fmicb.2023.1296008 PMC10658910

[vbag057-B10] Kobel CM , Leu A, Vera-Ponce de León A et al Protozoal populations drive system-wide variation in the rumen microbiome. Nat Commun 2025;16:6238. 40623969 10.1038/s41467-025-61302-2 PMC12234698

[vbag057-B11] Kobel CM , Merkesvik J, Tokvam Burgos IM et al Integrating host and microbiome biology using holo-omics. Mol Omics 2024;20:438–52. 38963125 10.1039/d4mo00017j

[vbag057-B12] Kwoji ID , Aiyegoro OA, Okpeku M et al Multi-omics’ data integration: applications in probiotics studies. NPJ Sci Food 2023;7:25. 37277356 10.1038/s41538-023-00199-x PMC10241933

[vbag057-B13] Langfelder P , Horvath S. WGCNA: an R package for weighted correlation network analysis. BMC Bioinformatics 2008;9:559. 19114008 10.1186/1471-2105-9-559 PMC2631488

[vbag057-B14] Layunta E , Buey B, Mesonero JE et al Crosstalk between intestinal serotonergic system and pattern recognition receptors on the Microbiota-Gut-Brain axis. Front Endocrinol (Lausanne) 2021;12:748254. 34819919 10.3389/fendo.2021.748254 PMC8607755

[vbag057-B15] Li G , Yu X, Portela Fontoura AB et al Transcriptomic regulations of heat stress response in the liver of lactating dairy cows. BMC Genomics 2023;24:410. 37474909 10.1186/s12864-023-09484-1 PMC10360291

[vbag057-B16] Ma N , Guo J, Li Z et al Disturbances of ruminal microbiota and liver inflammation, mediated by LPS and histamine, in dairy cows fed a High-Concentrate diet. Animals 2024;14:1495. 38791713 10.3390/ani14101495 PMC11117260

[vbag057-B17] Muller E , Shiryan I, Borenstein E. Multi-omic integration of microbiome data for identifying disease-associated modules. Nat Commun 2024;15:2621. 38521774 10.1038/s41467-024-46888-3 PMC10960825

[vbag057-B18] Munk K , Ilina D, Ziemba L et al Holomics - a user-friendly R shiny application for multi-omics data integration and analysis. BMC Bioinformatics 2024;25:93. 38438871 10.1186/s12859-024-05719-4 PMC10913680

[vbag057-B19] Nyholm L , Koziol A, Marcos S et al Holo-Omics: integrated Host-Microbiota multi-omics for basic and applied biological research. iScience 2020;23:101414. 32777774 10.1016/j.isci.2020.101414 PMC7416341

[vbag057-B20] Pereira AM , de Lurdes Nunes Enes Dapkevicius M, Borba AES. Alternative pathways for hydrogen sink originated from the ruminal fermentation of carbohydrates: which microorganisms are involved in lowering methane emission? Anim Microbiome 2022;4:5. 34991722 10.1186/s42523-021-00153-w PMC8734291

[vbag057-B21] Robinson JM , Cameron R. The holobiont blindspot: relating Host-Microbiome interactions to cognitive biases and the concept of the ‘’umwelt. Front Psychol 2020;11:591071. 33281689 10.3389/fpsyg.2020.591071 PMC7705375

[vbag057-B22] Rohart F , Gautier B, Singh A et al mixOmics: an R package for 'omics feature selection and multiple data integration. PLoS Comput Biol 2017;13:e1005752. 29099853 10.1371/journal.pcbi.1005752 PMC5687754

[vbag057-B23] Rossi A , Bellone A, Fokin SI et al Detecting associations between ciliated protists and prokaryotes with culture-independent single-cell microbiomics: a proof-of-concept study. Microb Ecol 2019;78:232–42. 30411190 10.1007/s00248-018-1279-9

[vbag057-B24] Rowland I , Gibson G, Heinken A et al Gut microbiota functions: metabolism of nutrients and other food components. Eur J Nutr 2018;57:1–24. 10.1007/s00394-017-1445-8 PMC5847071 28393285

[vbag057-B25] Shabat SK , Sasson G, Doron-Faigenboim A et al Specific microbiome-dependent mechanisms underlie the energy harvest efficiency of ruminants. ISME J 2016;10:2958–72. 27152936 10.1038/ismej.2016.62 PMC5148187

[vbag057-B26] Stepniewski TM , Mancini A, Ågren R et al Mechanistic insights into dopaminergic and serotonergic neurotransmission - concerted interactions with helices 5 and 6 drive the functional outcome. Chem Sci 2021;12:10990–1003. 34522296 10.1039/d1sc00749a PMC8386650

[vbag057-B27] Wang S , Giller K, Kreuzer M et al Contribution of ruminal fungi, archaea, protozoa, and bacteria to the methane suppression caused by oilseed supplemented diets. Front Microbiol 2017;8:1864. 29033916 10.3389/fmicb.2017.01864 PMC5626831

[vbag057-B28] Yang C , Rooke JA, Cabeza I et al Nitrate and inhibition of ruminal methanogenesis: microbial ecology, obstacles, and opportunities for lowering methane emissions from ruminant livestock. Front Microbiol 2016;7:132. 26904008 10.3389/fmicb.2016.00132 PMC4751266

[vbag057-B29] Yu G , Wang LG, Han Y et al clusterProfiler: an R package for comparing biological themes among gene clusters. OMICS 2012;16:284–7. 22455463 10.1089/omi.2011.0118 PMC3339379

[vbag057-B30] Zhao Y , Chang C, Long Q. Knowledge-Guided statistical learning methods for analysis of High-Dimensional -Omics data in precision oncology. JCO Precis Oncol 2019;3:PO.19.00018. 35100722 10.1200/PO.19.00018 PMC9797232

