# Supplementary material for: OmniCorr: an R-package for visualizing putative host-microbiome interactions using multi-omics data
Source: Bioinform Adv. 2026 Feb 17;6(1):vbag057. doi: 10.1093/bioadv/vbag057 (PMC12961270; doi:10.1093/bioadv/vbag057)
Supplement: vbag057_Supplementary_Data [file vbag057_supplementary_data.docx]

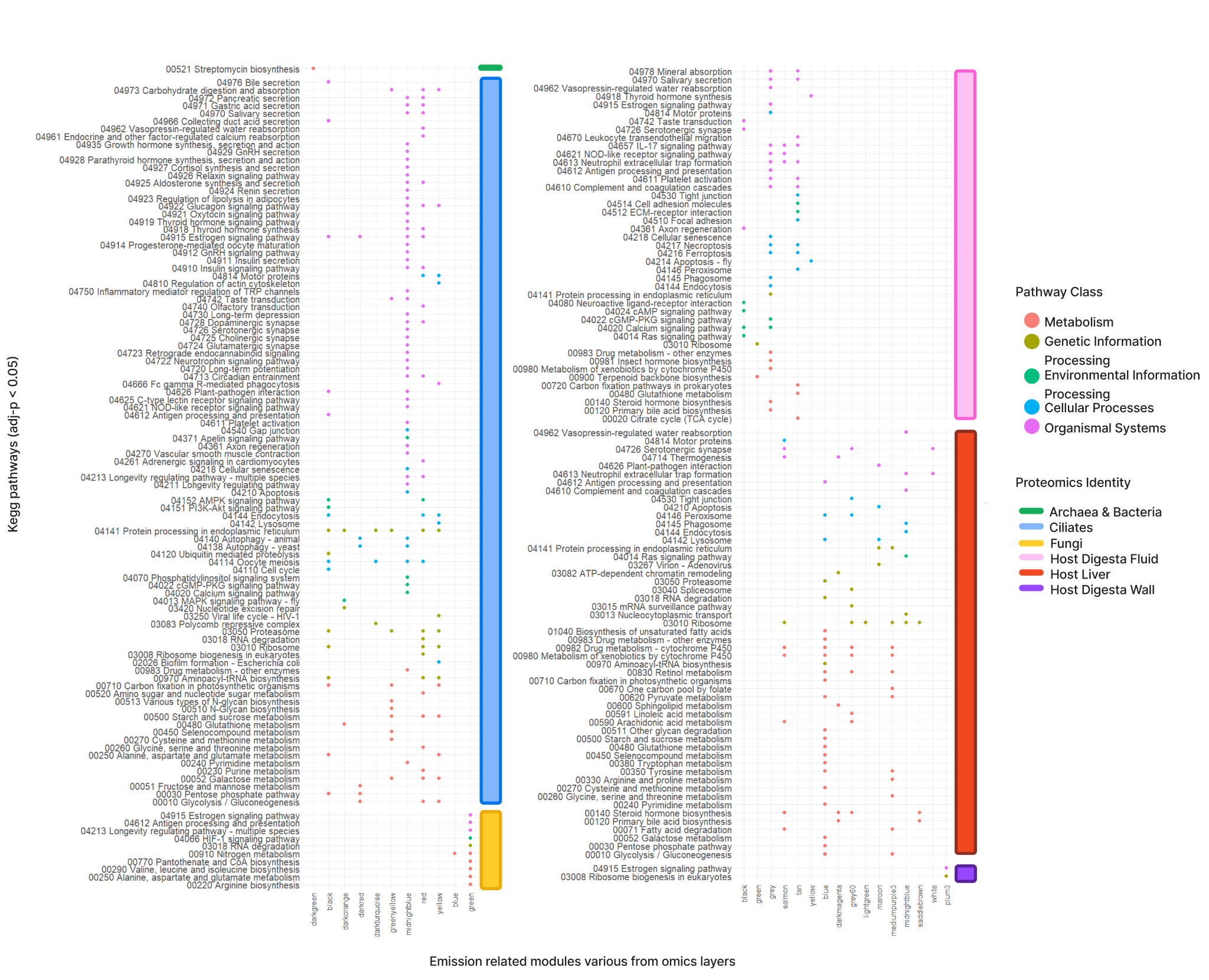


*Supplementary Figure 1: Modules with enriched KEGG pathway (adj. P-value < 0.05) identified across various omics layers.*

*
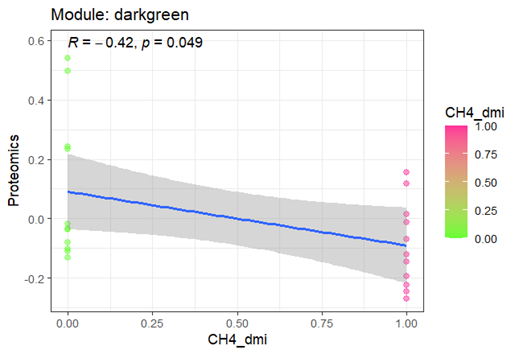
*

*Supplementary Figure 2: 22 proteins from the darkgreen modules with higher abundance in low methane emission cattles than high methane emission cattles.*

**

*Supplementary Figure 3: Multi-omics factor analysis (MOFA) delineates the sources of heterogeneity between species.*

A. Bar plot showing the total variance explained (%) by the MOFA model across the different data modalities (views) included in the analysis. Each bar represents one view: Archaea & Bacteria, Ciliates, Fungi, Host (Digesta Fluid), Host (Digesta Wall), and Host (Liver). The height of each bar indicates how much of the total variability in that dataset is captured by the MOFA factors. This plot summarizes how well the model explains variation within each omics layer, highlighting which datasets contribute most or least to the inferred factors.

B. Heatmap showing the percentage of variance explained (R^2^) by each factor (rows) across the six modalities (columns). Factors 1 and 3 capture coordinated variation across multiple views, whereas Factors 4 and 5 identify heterogeneity predominantly driven by host datasets.

C. Association analysis between factor values and sample metadata covariates, including methane emission intensity (ch4_g_kg_dmi). Circles represent Pearson correlation coefficients between each factor (rows) and covariate (columns), with blue indicating positive and red indicating negative correlations. None of the associations between MOFA factors and methane emission intensity are statistically significant (p-value > 0.05), indicating that methane output variation is not captured by these latent factors.

D. Enrichment of dark green module proteins across MOFA factors. Fisher’s exact test assesses whether proteins from the WGCNA dark green module (identified during the OmniCorr pipeline) are enriched among the top 20% of proteins contributing to each MOFA factor. The x-axis shows the -log_10_(P-value), and the red dashed line marks the significance threshold (p = 0.05). MOFA factors 6 and 8 display significant enrichment, indicating that dark green module proteins are associated with the biological variation captured by these factors.
